# Separation of current density and electric field domains caused by nonlinear electronic instabilities

**DOI:** 10.1038/s41467-018-04452-w

**Published:** 2018-05-23

**Authors:** Suhas Kumar, R. Stanley Williams

**Affiliations:** Hewlett Packard Labs, 1501 Page Mill Rd, Palo Alto, CA 94304 USA

## Abstract

In 1963 Ridley postulated that under certain bias conditions circuit elements exhibiting a current- or voltage-controlled negative differential resistance will separate into coexisting domains with different current densities or electric fields, respectively, in a process similar to spinodal decomposition of a homogeneous liquid or disproportionation of a metastable chemical compound. The ensuing debate, however, failed to agree on the existence or causes of such electronic decomposition. Using thermal and chemical spectro-microscopy, we directly imaged signatures of current-density and electric-field domains in several metal oxides. The concept of local activity successfully predicts initiation and occurrence of spontaneous electronic decomposition, accompanied by a reduction in internal energy, despite unchanged power input and heat output. This reveals a thermodynamic constraint required to properly model nonlinear circuit elements. Our results explain the electroforming process that initiates information storage via resistance switching in metal oxides and has significant implications for improving neuromorphic computing based on nonlinear dynamical devices.

## Abstract (web summary)

The usefulness of metal-oxide memristors for memory and brain-inspired computing applications arises from their electronic instabilities, whose details remain limited. In this work, the authors analyze electronic decompositions in several metal oxides, providing new insights for device modeling.

## Introduction

Electronic instabilities in materials, such as negative differential resistance (NDR), have seen a surge of recent interest because of their potential applications in storage-class memory^1–3^ and bio-inspired neuromorphic computing^4–7^. By invoking symmetry-breaking via maximum entropy creation, Ridley^8^ predicted the formation of two different coexisting domains of current-density channels when a material that exhibits current-controlled NDR is voltage biased in an unstable operating regime. The proposed mechanism was questioned by Landauer^9,10^, who suggested that minimum heat generation and interaction dynamics between coexisting stable states were key to understanding electronic domain decomposition. Schöll demonstrated that domain decomposition and chaos were possible for the specific case of hot electrons interacting with impurity levels in semiconductors^11,12^. However, no consensus on a general mechanism has arisen in this debate, and in particular the question of how to distinguish between different, and possibly coexisting, steady states in a nonlinear electronic system remains unanswered^12–17^. There has been a parallel debate on whether state transitions, such as resistance switching in metal oxides, are initiated by electronic or thermal instabilities^12,15,16,18,19^. With a few exceptions^20^, most recent models of NDR and related instabilities have ignored the possibility of decomposition into co-existing domains^1,6,21–27^.

Here we show from experimental imaging and an extended nonlinear dynamics model that an electronic system biased within an unstable operating region undergoes spontaneous decomposition into coexisting stable and distinct domains, even when the power input and heat generated by the decomposed states are identical to those of the uniform unstable state. However, the increase of the internal energy of the nonlinear material from ambient conditions is lower for the decomposed compared to the uniform unstable system. We illustrate these concepts via current-density decomposition during current-controlled NDR, and the co-existence of current-density and electric-field decomposition during the temperature-controlled instability of a Mott transition. We utilize the concept of local activity^28,29^ as the framework to understand the electronic instabilities and the amplification mechanism of local thermal fluctuations that drives these systems to the lowest internal energy steady state.

## Results

### Current-controlled NDR from temperature-activated transport

**Fig. 1:**
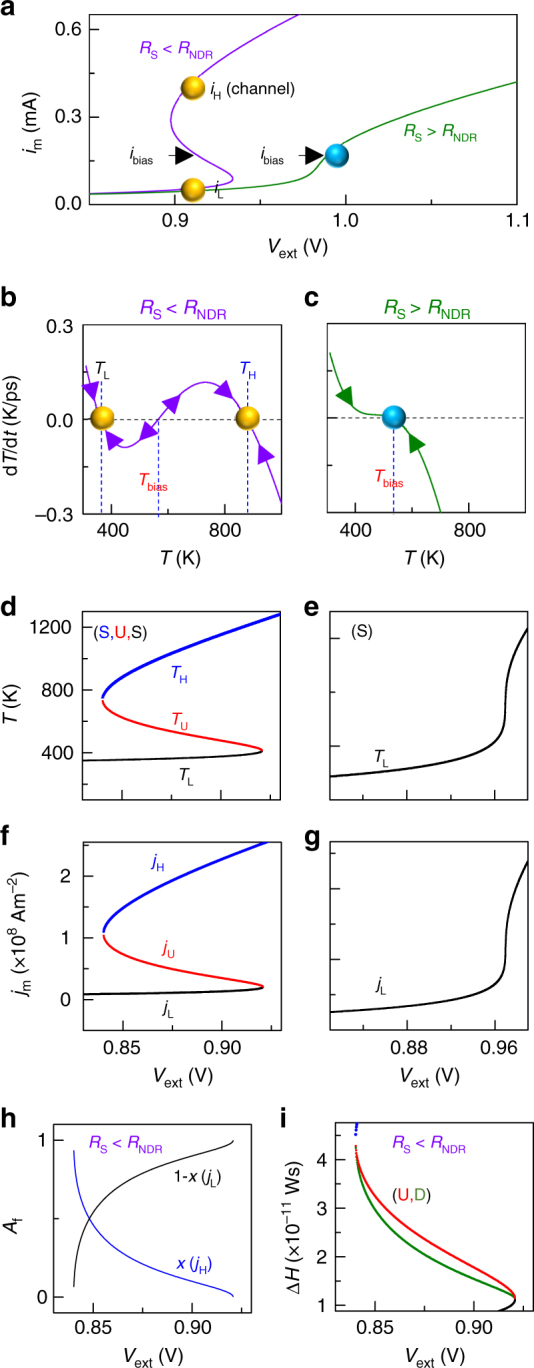
Current-controlled negative differential resistance (NDR). **a** *i*_m_–*V*_ext_ plots from Eqs. (1–2) for two different cases: *R*_S_ < *R*_NDR_ and *R*_S_ > *R*_NDR_. Bias current (*i*_bias_) and the high and low stable currents (*i*_H_ and *i*_L_) are marked. **b** d*T*/d*t* vs. *T* for the case of *R*_S_ < *R*_NDR_ for an applied voltage of 0.91 V. Temperatures corresponding to *i*_bias_, *i*_H_ and *i*_L_ are marked (*T*_bias_, *T*_H_ and *T*_L_). **c** d*T*/d*t* vs. *T* for the case of *R*_S_ > *R*_NDR_ for an applied voltage of 0.96 V. **d** Stable (‘S’) (*T*_H_ and *T*_L_) and unstable (‘U’) (*T*_bias_) temperatures corresponding to the steady-states for different applied *V*_ext_. **e** Same as **d** for the case of *R*_S_ > *R*_NDR_. **f** Stable (*j*_H_ and *j*_L_) and unstable (*j*_U_) current densities corresponding to the steady-states for different applied *V*_ext_. **g** Same as **f** for the case of *R*_S_ > *R*_NDR_. **h** Area fractions (*A*_f_) of the two current-density states (*x* and 1 − *x*) plotted against *V*_ext_ for *R*_S_ < *R*_NDR_. **i** Δ*H* of the active layer vs. *V*_ext_ for the unstable (‘U’) and decomposed (‘D’) states for *R*_S_ < *R*_NDR_

A current-controlled NDR in materials such as the oxides of Ti, Hf, Nb, Ta, etc. results from a highly non-linear and activated transport mechanism that enables positive feedback by Joule heating, leading to an increase in current accompanied by a decrease in device voltage^1,21,30^. When the power dissipated and heat removed are not at steady state, such a system can be modeled by a memristor formalism with the temperature *T* as the state variable^6,31^. We reprise one such model using a modified 3D Poole–Frenkel equation^1^ as the quasi-static equation for NbO_2_ connecting the memristor current, *i*_m_, and voltage, *v*_m_:

1 $${\it{i}}_{\mathrm{m}} = \left[ {{\it{\sigma }}_0{\mathrm{e}}^{ - \frac{{0.301}}{{2{\it{k}}_{\mathrm{B}}{\mathrm{T}}}}}{\it{A}}\left\{ {\left( {\frac{{{\it{k}}_{\mathrm{B}}{\it{T}}}}{{{\it{\omega }}\sqrt {{\it{v}}_{\mathrm{m}}} }}} \right)^2\left( {1 + \left( {\frac{{{\it{\omega }}\sqrt {{\it{v}}_{\mathrm{m}}/{\it{d}}} }}{{{\it{k}}_{\mathrm{B}}{\it{T}}}} - 1} \right){\mathrm{e}}^{\frac{{{\it{\omega }}\sqrt {{\it{v}}_{\mathrm{m}}/{\it{d}}} }}{{{\it{k}}_{\mathrm{B}}{\it{T}}}}}} \right)+ \frac{1}{{2{\it{d}}}}} \right\}} \right]{\it{v}}_{\mathrm{m}}{\mathrm{,}}$$

where *A* is the lateral device area; *d* the thickness of the oxide; *k*_B_ the Boltzmann constant (8.617 × 10^−5^ eV); *T* the absolute temperature; and *ω* and *σ*_*0*_ are material constants, described elsewhere^1^. The equation determining the device dynamics is just Newton’s law of cooling:

2 $$\frac{{{\mathrm{d}}{\it{T}}}}{{{\mathrm{d}}{\it{t}}}} = \frac{{{\it{i}}_{\mathrm{m}}{\it{v}}_{\mathrm{m}}}}{{{\it{C}}_{{\mathrm{th}}}^{{\mathrm{sys}}}}} - \frac{{{\it{T}} - {\it{T}}_{{\mathrm{amb}}}}}{{{\it{C}}_{{\mathrm{th}}}^{{\mathrm{sys}}}{\it{R}}_{{\mathrm{th}}}}}{\mathrm{,}}$$

where *T*_amb_ is the ambient temperature (300 K); ${\it{C}}_{{\mathrm{th}}}^{{\mathrm{sys}}}$ is the thermal capacitance of the system comprising the active NbO_2_ layer, the device structure, and its thermal environment^32^; and *R*_th_ is the effective thermal resistance of the entire device embedded in its ambient (1.4 × 10^6^ KW^−1^). Estimates for the thermal constants are discussed elsewhere^32,33^. For an applied external voltage *V*_ext_, the steady state *i*_m_–*V*_ext_ relationship (Fig. 1a) obtained by solving Eqs. (1 and 2) with $\frac{{{\mathrm{d}}{\it{T}}}}{{{\mathrm{d}}{\it{t}}}} = 0$ and *V*_ext_ = *v*_m_ + *i*_m_*R*_s_ exhibits NDR when the series resistance (*R*_S_) is less than *R*_NDR_, where *R*_NDR_ is defined as the absolute magnitude of the largest negative slope of the *i*_m_–*v*_m_ curve within the NDR region (about 350 Ω). However, when *R*_S_ = 360 Ω is added to the circuit, the electrical behavior is stabilized^8,34^ (green curve). The origins of electronic decomposition and subsequent stabilization within the circuit by adding *R*_S_ are revealed by plotting the dynamical route of the system ($\frac{{{\mathrm{d}}{\it{T}}}}{{{\mathrm{d}}{\it{t}}}}$ against *T* by parametrically sweeping *T*, after combining Eqs. (1 and 2))^35,36^. In the case of *R*_S_ less than *R*_NDR_, at an applied voltage within the NDR region, there are three zero-crossings of the dynamical route (Fig. 1b), corresponding to three possible steady-state temperatures $\left( {\frac{{{\mathrm{d}}{\it{T}}}}{{{\mathrm{d}}{\it{t}}}} = 0} \right)$. The arrows on the dynamical route are determined by whether $\frac{{{\mathrm{d}}{\it{T}}}}{{{\mathrm{d}}{\it{t}}}} > 0$ (*T* increases) or $\frac{{{\mathrm{d}}{\it{T}}}}{{{\mathrm{d}}{\it{t}}}}$ < 0 (*T* decreases). Upon a small perturbation in *T*, stable steady-states will return to the original state (arrows pointing inwards), while unstable steady-states do not return (arrows pointing outwards). A possible state evolution when the system is biased at an unstable steady state is to decompose into coexisting physical domains corresponding to the two stable steady-states on either side of the unstable state. The unstable current levels within the NDR region can be accessed through transients, but are typically not accessed through voltage sweeps (Supplementary Fig. 1). For the circuit with *R*_S_ greater than *R*_NDR_ (Fig. 1c), there is only one stable steady-state (blue sphere), and thus no regions of instability or NDR (also see Supplementary Fig. 2). In other words, operation by a current source, or a voltage source with a sufficiently large *R*_S_, will lead to stabilization and no decompositions. Thus the necessary conditions for electronic domain decomposition under a given bias are: (i) the presence of at least one unstable and two stable steady states, and (ii) experimental access to an unstable steady state. We determine the lower and higher stable (*T*_L_ and/or *T*_H_, respectively) and unstable (*T*_U_), if any, steady-state value(s) of *T* through the dynamical route maps corresponding to every *V*_ext_ (Fig. 1d,e). Using this ‘phase plot’ of *T* vs. *V*_ext_, and the functional relationship between *T* and *i*_m_ (Supplementary Fig. 3), we calculated the current densities (*j*_m_) corresponding to every *V*_ext_, which reveals that for a range of *V*_ext_, there are a pair of stable current densities (*j*_H_ and *j*_L_) along with an unstable current density (*j*_U_) (Fig. 1f, g, Supplementary Fig. 4). The decomposition occurs via the formation of two adjacent current density domains,

3 $${\it{j}}_{\mathrm{U}}.{\it{A}} = {\it{j}}_{\mathrm{L}}.({\it{A}} - {\it{x}}.{\it{A}}) + {\it{j}}_{\mathrm{H}}.({\it{x}}.{\it{A}}),$$

where *A* is the lateral device area and *x* is the fraction of *A* carrying current density *j*_H_. The values of *x* and 1 − *x* can be obtained from Eq. (3) for different *V*_ext_ (Fig. 1h) in the NDR region. In order to estimate the relative internal energies of the decomposed (D) and unstable (U) configurations, we calculated the change in enthalpy of the active oxide film (Δ*H*) from ambient conditions to the steady states (also see Supplementary Note 1):

4a $${\mathrm{\Delta }}{\it{H}}_{\mathrm{D}} = (1 - {\it{x}}) \cdot {\mathrm{\Delta }}{\it{H}}_{\mathrm{L}} + {\it{x}}.{\mathrm{\Delta }}{\it{H}}_{\mathrm{H}}$$

4b $${\mathrm{\Delta }}{\it{H}}_{\mathrm{D}} = \left( {1 - {\it{x}}} \right) \cdot \left[ {\mathop {\smallint }\limits_{{\it{T}}_{{\mathrm{amb}}}}^{{\it{T}}_{\mathrm{L}}} {\it{C}}_{{\mathrm{th}}}^{{\mathrm{act}}}\left( {\it{T}} \right){\mathrm{d}}{\it{T}}} \right] + {\it{x}} \cdot \left[ {\mathop {\smallint }\limits_{{\it{T}}_{{\mathrm{amb}}}}^{{\it{T}}_{\mathrm{H}}} {\it{C}}_{{\mathrm{th}}}^{{\mathrm{act}}}\left( {\it{T}} \right){\mathrm{d}}{\it{T}}} \right]$$

and

4c $${\mathrm{\Delta }}{\it{H}}_{\mathrm{U}} = \mathop {\smallint }\limits_{{\it{T}}_{{\mathrm{amb}}}}^{{\it{T}}_{\mathrm{U}}} {\it{C}}_{{\mathrm{th}}}^{{\mathrm{act}}}\left( {\it{T}} \right){\mathrm{d}}{\it{T}}{\mathrm{,}}$$

respectively, where ${\it{C}}_{{\mathrm{th}}}^{{\mathrm{act}}}$ is the thermal capacitance of only the active NbO_2_ layer within the device structure, Δ*H*_L_ and Δ*H*_H_ are the enthalpy changes from ambient corresponding to regions with temperatures *T*_L_ and *T*_H_, respectively, and *T*_amb_ is the ambient temperature (300 K). Figure 1i shows that the decomposed configuration has a lower enthalpy at steady state. Criteria based on maximization of entropy or minimizing total heat generated fail to distinguish between the two configurations^8,10,15^, because the input power and heat released by the two possibilities are identical.

**Fig. 2:**
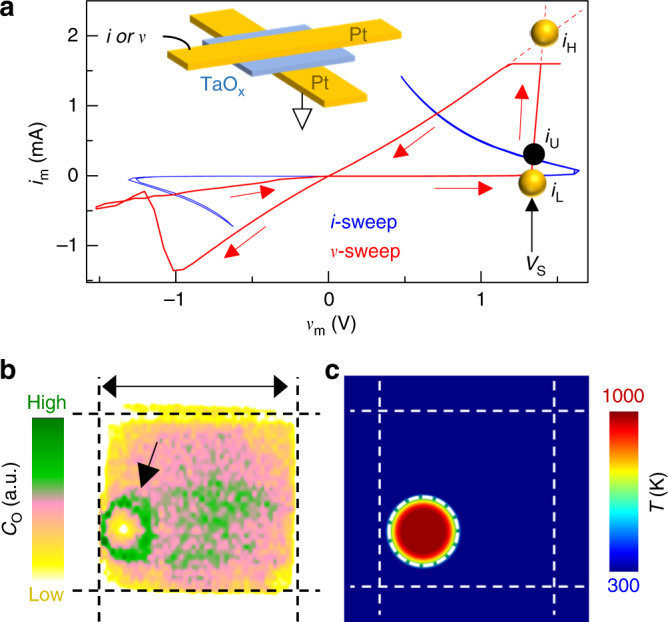
Current-density-decomposition as a precursor to electroforming in memristors. **a** An *i*-sweep (blue) on a previously un-operated crosspoint tantalum oxide memristor clearly showing reversible current-controlled NDR. A *v*-sweep (red) following the *i*-sweep on the same device, revealing non-volatile switching. Positive *v*-sweep was applied first, with the abrupt non-volatile switching occurring at a voltage value of *V*_S_. Inferred stable (*i*_H_ and *i*_L_) and unstable (*i*_U_) current levels are marked on the experimental data. *i*_H_ was extrapolated from the total electrode resistance since this is the only internal current compliance capable of arresting transient current overshoots. (**a**, inset) schematic of the crosspoint device structure. **b** Lateral oxygen concentration (*C*_O_) map of a different but nominally identical device to that shown in **a** after several non-volatile switching events, obtained by transmission synchrotron x-ray spectromicroscopy. The ring-like feature (indicated by a black arrow) is caused by a radial oxygen concentration gradient. Dashed lines mark the edges of the electrodes. **c** A simulated temperature (*T*) profile for a decomposed current density calculated from the measured current/voltage levels during operation, with a common scale displayed in **c**. The common scale bar in **b** is 2 µm. Additional analysis shown in Supplementary Fig. 6

We now demonstrate that current-density decompositions can be the precursor to non-volatile resistance switching in a TaO_x_ memristor, which initially exhibited reversible current-controlled NDR in its *i*_m_*–v*_m_ behavior when powered by a quasi-static current source (blue curve, Fig. 2a, see Methods)^37^. However, when subsequently operated with a quasi-static voltage source, it displayed an abrupt jump in current followed by non-volatile resistive switching (red curve, Fig. 2a). This behavior was caused by a decomposition that created a high-current density and thus high-temperature region in the oxide. The large temperature gradient induced thermophoresis and subsequent Fick diffusion of oxygen, creating radial chemical and electrical conduction gradients in the oxide that persisted even after the power was withdrawn (Fig. 2b, c)^38,39^, thus providing a channel for non-volatile resistance switching. Minimization of the interface energy between the two coexisting domains yields a cylindrical channel of one current density embedded in the other, explaining the circular feature in the oxygen map of Fig. 2b. To further support this interpretation, we used the measured NDR and subsequent resistive switching behavior of a memristor^38^ to identify the stable and unstable current levels (*i*_H_, *i*_L_ and *i*_U_ in Fig. 2a, with *i*_U_ limited by the device’s steady-state internal resistance). From these, we calculated the expected area of the high current density domain using Eq. (3) (dashed circle, Fig. 2c), which agrees with the observed channel in Fig. 2b. Thus, we conclude that the voltage-sourced electroforming process for transition metal oxides is consistent with a current-density decomposition (illustrated in Supplementary Fig. 5) that produces a much higher temperature channel than its surroundings, which in turn induces the oxygen stoichiometry changes responsible for nonvolatile electrical switching. The formation of a channel can be avoided by using a current source to initially power the sample, which will result in uniform current flow and heating of the device^32^.

### Temperature-controlled instability of a Mott transition

**Fig. 3:**
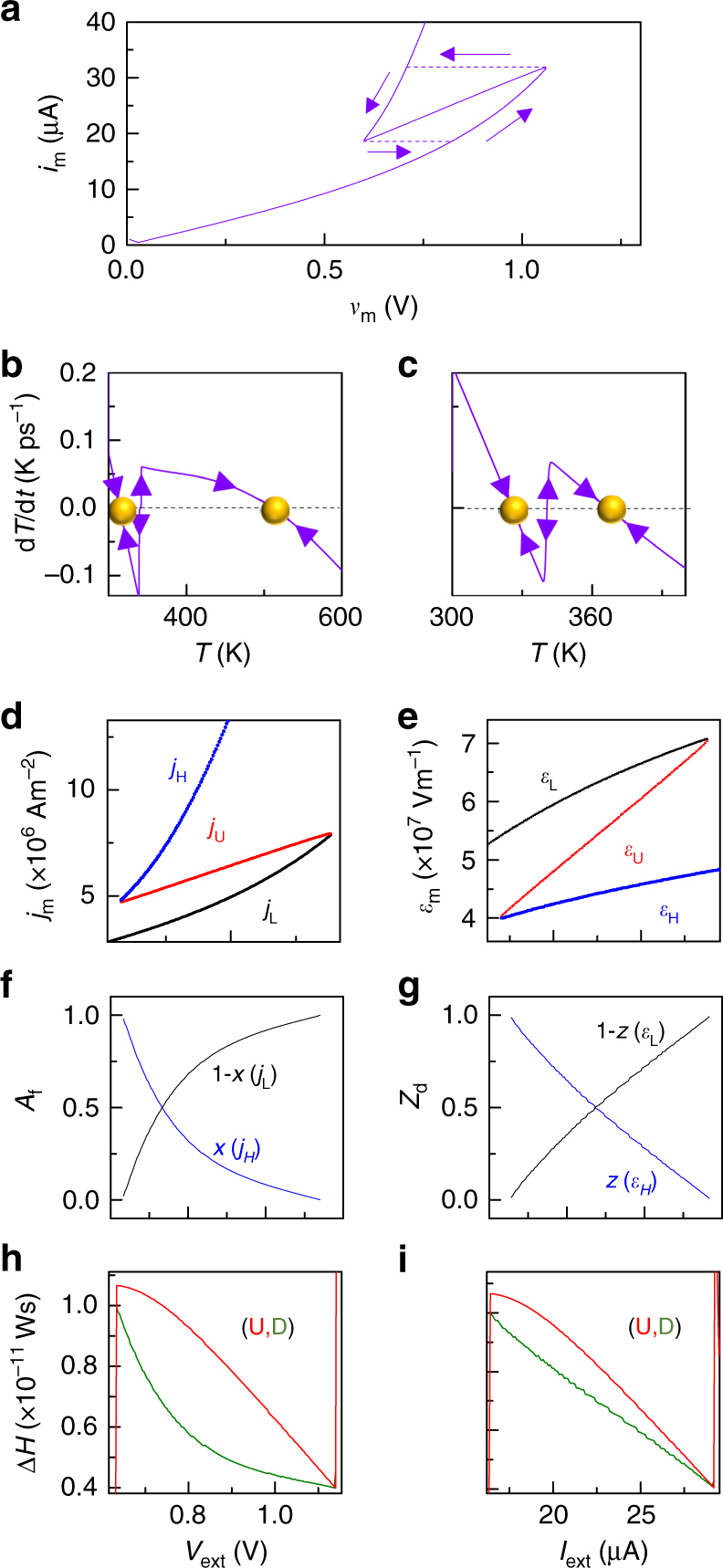
Temperature-controlled instability due to a Mott transition. **a** *i*_m_*–v*_m_ plots from Eqs. (1) and (2) with the Mott transition at 340 K parameterized within *R*_th_, representing VO_2_. Solid line: parametric T-sweep. Dashed line: *i*_m_-sweep. **b** d*T*/d*t* vs. *T* for an applied external voltage of 0.7 V. **c** d*T*/d*t* vs. *T* for an applied external current of 25 µA. Yellow spheres indicate stable steady-states. **d** Stable (*j*_H_ and *j*_L_) and unstable (*j*_U_) current densities corresponding to the steady-states for different applied *V*_ext_. **e** Stable (*ε*_H_ and *ε*_L_) and unstable (*ε*_U_) electric-fields corresponding to the steady-states for different applied external current *I*_ext_. **f** Area fractions (*A*_f_) of the two decomposed current-density states (*x* and 1 − *x*) plotted against *V*_ext_. **g** Thickness fractions (*Z*_d_) containing the two decomposed states (*z* and 1 − *z*) plotted against *I*_ext_. **h** Δ*H* vs. *V*_ext_ for the unstable (‘U’) and decomposed (‘D’) current-density states. **i** Δ*H* vs. *I*_ext_ for the unstable and decomposed electric-field states

We next consider a temperature-controlled instability in which both the current and voltage decrease as the temperature of the system is increased (Fig. 3a), which is caused by a Mott transition, observed for example in VO_2_ and NbO_2_^4,32,40^. In order to model this behavior, we still used Eq. (1) to describe electrical transport, but we introduced an abrupt increase^32,41^ in *R*_th_ at the transition temperature *T*_MIT_ (chosen to be 340 K to represent VO_2_) in Eq. (2), which produces the *i*_m_*–v*_m_ plot in Fig. 3a. Further, to account for the latent heat of the Mott transition, we utilized a spiked-increase in ${\it{C}}_{{\mathrm{th}}}^{{\mathrm{act}}}$ as a function of *T* at *T*_MIT_ (see Methods, Supplementary Fig. 7). The dynamical routes of the system corresponding to operation separately by a current source or a voltage source within the region of instability are each characterized by an unstable steady-state flanked by stable steady-states (Fig. 3b, c), revealing that both bias conditions will produce electronic decompositions. We repeated the dynamical analysis presented in Fig. 1 to obtain a plot of *j*_m_ vs. *V*_ext_ (Fig. 3d, Supplementary Fig. 8), for the case of using an external voltage source with *R*_S_ = 0. For the case of using an external current source, Fig. 3e shows the plot of the electric field (*ε*_m_) vs. applied current (*I*_ext_). In each case, there are three steady-state current densities or electric fields (two stable and one unstable). We represent the decomposition in the electric field as

5 $${\it{\varepsilon }}_{\mathrm{U}}.{\it{d}} = {\it{\varepsilon }}_{\mathrm{L}}.({\it{d}} - {\it{z}}.{\it{d}}) + {\it{\varepsilon }}_{\mathrm{H}}.({\it{z}}.{\it{d}}),$$

where *ε*_U_ is the unstable uniform electric field, *ε*_H_ and *ε*_L_ are stable high and low electric fields, respectively, *d* is the thickness of the oxide film, and *z* is the fraction of *d* containing the electric field *ε*_H_. Using Eqs. (3 and 5) on the data in Fig. 3d, e, we calculate the area fractions (*A*_f_) and the thickness fractions (*Z*_d_) for the current-density and the electric-field decompositions, respectively (Fig. 3f,g). For the case of electric-field decompositions, we calculate Δ*H* using Eq. (4) replacing *x* with *z*, while *T*_H_ and *T*_L_ correspond to *ε*_H_ and *ε*_L_, respectively. Δ*H* for the unstable state is calculated as ${\mathrm{\Delta }}{\it{H}}_{\mathrm{U}} = \mathop {\smallint }\limits_{{\it{T}}_{{\mathrm{amb}}}}^{{\it{T}}_{\mathrm{U}}} {\it{C}}_{{\mathrm{th}}}^{{\mathrm{act}}}({\it{T}}){\mathrm{d}}{\it{T}}$, where *T*_U_ corresponds to *ε*_U_. Enthalpy calculations for both decompositions (Fig. 3h, i) show that the decomposed states have the lower Δ*H*. A temperature-controlled instability caused by a Mott transition will exhibit decomposed current-density channels during voltage source operation and electric-field fragments during current source operation.

**Fig. 4:**
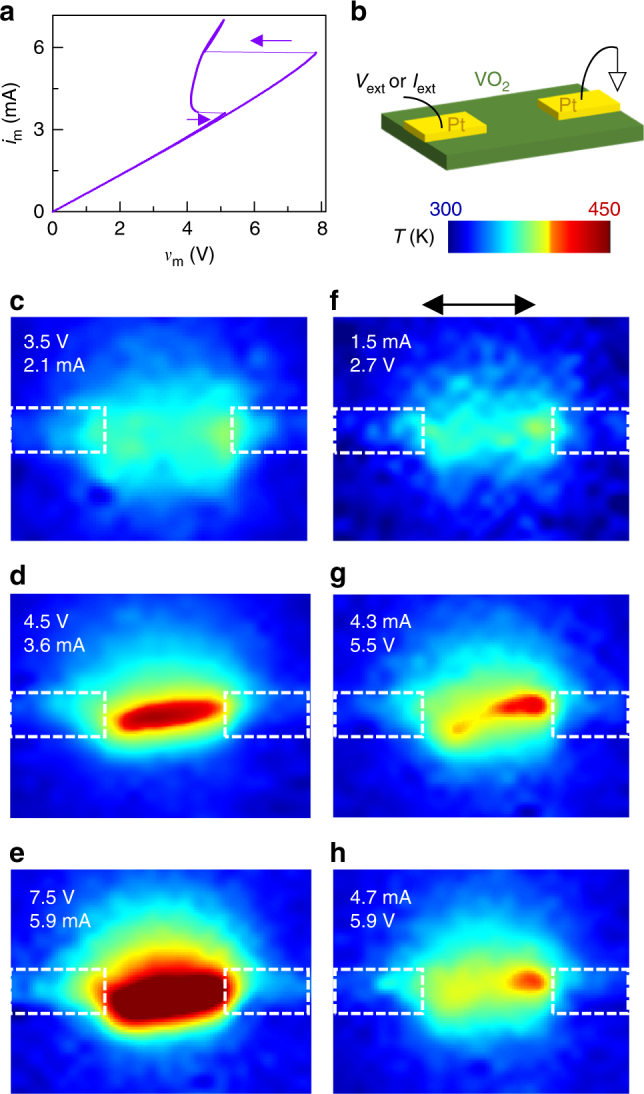
Current-density- and electric-field-decompositions in VO_2_. **a** Experimentally measured *i*_m_*–v*_m_ plot from a lateral VO_2_ thin film with electrodes separated by 16 µm. **b** Schematic of the device structure. **c**–**e** Temperature (*T*) maps of the VO_2_ film with applied voltages (from a voltage source), along with the measured currents noted. **f**–**h** Temperature maps of the same device with applied currents (from a current source), along with the measured voltages noted. **c**–**h** were measured on a device that was different from but identical in geometry and composition to the one that produced data in **a**. Common scale bar in **f** is 16 µm. Additional analysis shown in Supplementary Figs 9–10

We experimentally demonstrate the above using VO_2_ (see Methods), a Mott insulator with a temperature-controlled instability^40^, for current flow in the plane of a thin film to visualize the field domains. A *i*_m_*–v*_m_ curve measured using a current source exhibits a pinched hysteresis (Fig. 4a) due to the Mott transition, which appears as a pair of sharp NDR transitions. Figure 4c–h displays in-operando blackbody emission temperature maps of a lateral VO_2_ device with metallic electrodes deposited on top of a thin VO_2_ film (device fabrication and measurements are detailed elsewhere)^40,42^. When powered by a voltage source near the unstable region, a high current-density channel, as revealed by the high-temperature map, connects the two electrodes (Fig. 4c–e). However, when operated with a current source within the unstable region, high-temperature domains appear (Fig. 4f–h) that do not connect the electrodes. This data qualitatively supports the model presented in Fig. 3. Notably, the high-temperature (above *T*_MIT_) and low-temperature (below *T*_MIT_) domains correspond to the regions of lower and higher power dissipation, respectively, in the *i*_m_*–v*_m_ curve in Fig. 4a. This counter-intuitive result arises because of the increase^32,41^ in the thermal resistance *R*_th_ observed at *T*_MIT_ (Supplementary Fig. 9).

### Dual current- and temperature-controlled instabilities

**Fig. 5:**
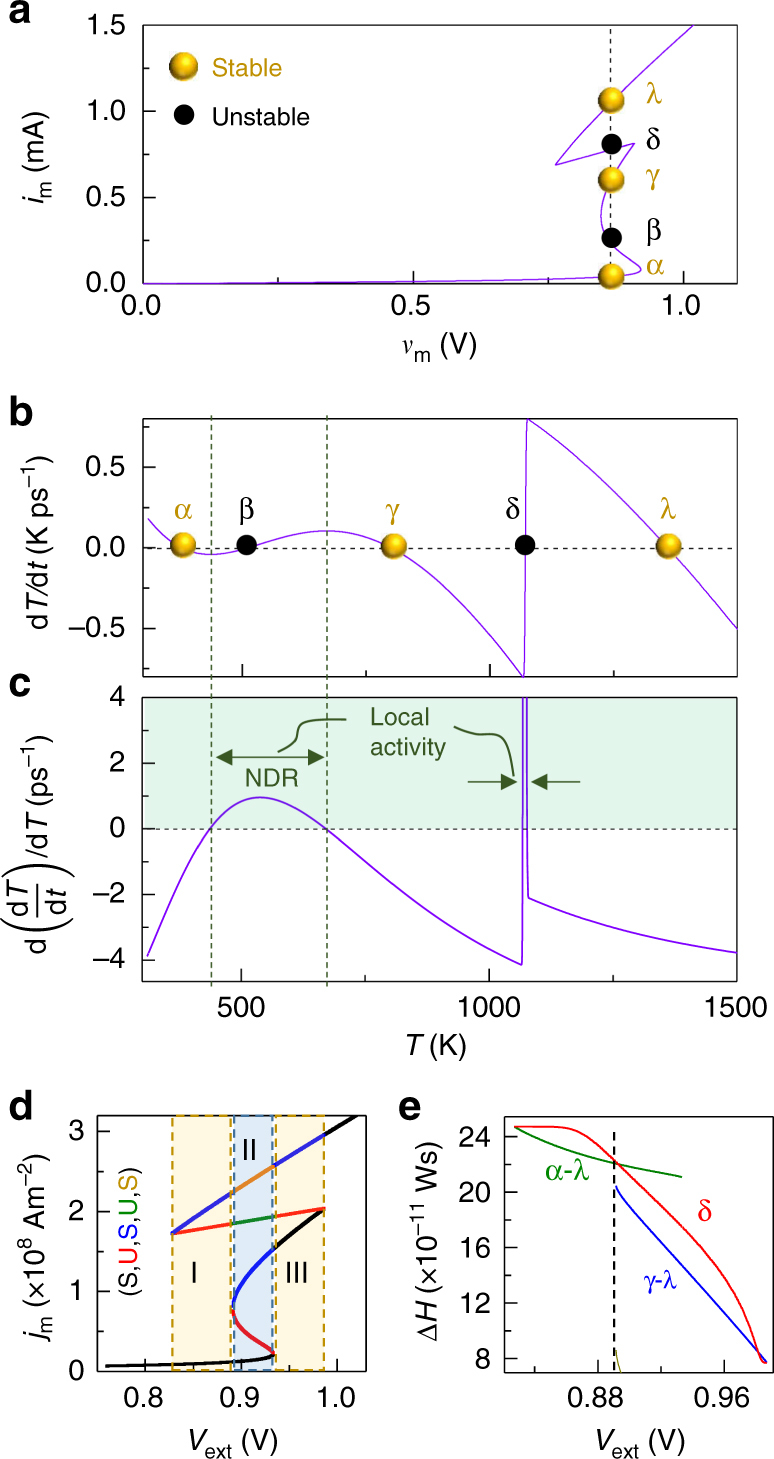
Current-controlled and temperature-controlled instabilities representing NbO_2_. **a** *i*_m_*–v*_m_ plot obtained by solving Eqs. (1) and (2) with a Mott transition at 1070 K described by *R*_th_. **b** The dynamical route map d*T*/d*t* vs. *T* for an applied external voltage of 0.91 V. Yellow and black spheres indicate stable and unstable steady-states, respectively. α*–*λ are steady-state points for an applied external voltage of 0.91 V. **c** $\frac{{\mathrm{d}}}{{{\mathrm{d}}{\it{T}}}}\left( {\frac{{{\mathrm{d}}{\it{T}}}}{{{\mathrm{d}}{\it{t}}}}} \right)$ plotted against *T*. Green shaded region indicates positive ordinate values where local activity exists. Temperature range of negative differential resistance (NDR) is also marked. **d** Stable (‘S’) and unstable (‘U’) current densities corresponding to the steady-states for different applied *V*_ext_, color coded with the legend. Dashed vertical lines separate regions I–III with different sets of steady-states. **e** Δ*H* plotted against *V*_ext_ for the unstable state δ and two possible decomposed configurations α–λ and γ*–*λ. Dashed black line indicates the voltage at which Δ*H* of γ−λ is lower than that of α–λ. Extended data shown in Supplementary Fig. 11

Having separately analyzed current-controlled NDR (similar to that in TaO_x_, TiO_x_, etc.) and a Mott transition (e.g., that in VO_2_), we now present the full example of NbO_2_, which exhibits both instabilities. This was modeled by using Eq. (1) to describe transport and Eq. (2) for the Mott transition^43,44^ with *T*_MIT_ = 1070 K. The resultant *i*_m_*–v*_m_ curve for *R*_S_ = 0 Ω is shown in Fig. 5a, and a voltage bias within the range containing both instabilities intersects the curve at five distinct points (α–λ). A plot of the dynamical route (Fig. 5b) at this bias reveals that two of the steady-states are unstable, with each flanked by a pair of stable steady-states. A plot of $\frac{{\mathrm{d}}}{{{\mathrm{d}}{\it{T}}}}\left( {\frac{{{\mathrm{d}}{\it{T}}}}{{{\mathrm{d}}{\it{t}}}}} \right)$ vs. *T* reveals the change in state equation of $\frac{{{\mathrm{d}}{\it{T}}}}{{{\mathrm{d}}{\it{t}}}}$ for a small perturbation in *T* (Fig. 5c). If this quantity is positive, it indicates amplification of the rate of temperature change for small perturbations in *T*, which is a signature of local activity^28,45^. This quantity also serves as a criterion for verifying local activity and the presence of associated NDR and/or other instabilities.

As demonstrated in Fig. 5c, both the current-controlled NDR and the Mott-transition instability are regions of local activity. Further, in order to analyze the possible current-density decompositions occurring within different operating regions, we repeat the exercise detailed in Fig. 1 to obtain a *j*_m_ vs. *T* plot (Fig. 5d). Here we see three regions (I–III) of bias where region II contains three stable steady-states while regions I and II contain two each. A potential  ternary decomposition involving a combination of all three stable steady-states within region II has a higher enthalpy than the component decompositions and is thus unstable; see Methods. We consider decompositions from state δ into α and λ, and separately into γ and λ. The enthalpy increases of the two possible decompositions and that for the unstable state δ (Fig. 5e, obtained using a procedure similar to that established in Fig. 1 and Eqs (3 and 4)), reveals that in region I, the α–λ decomposition has the lowest Δ*H*, while in regions II and III, the γ*–*λ decomposition has the lowest Δ*H*. A transition from one decomposed state to another would in general involve switching between different channels.

**Fig. 6:**
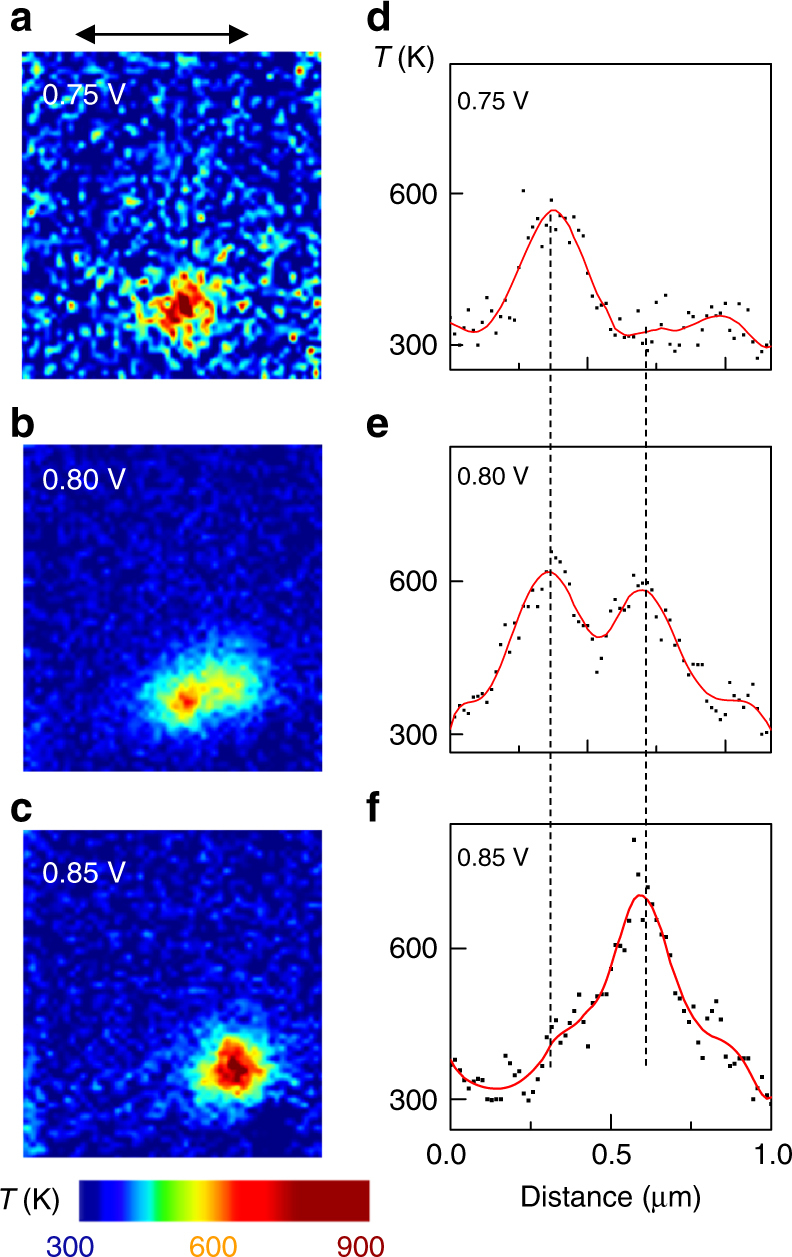
Experimental data for both cross-over decompositions and local activity. **a**–**c** Temperature maps of a crosspoint NbO_2_ device with different applied voltages (from a voltage source), as marked. **d**–**f** 1-dimensional temperature (*T*) profiles across the high-temperature channels in **a**. Scatter points are raw data and red solid curve is smoothed data. Common scale bar in **a** is 1 µm. Extended data are shown in Supplementary Fig. 12

We experimentally imaged micrometer-sized NbO_2_ crosspoint devices (see Methods, Supplementary Figs. 13–15), in which current flow is normal to the plane of the device, using in operando time-multiplexed thermoreflectance (device structures, the technique and measurements are detailed elsewhere)^32,46^. The temperature maps presented in Fig. 6 are averages of more than 1000 sequential maps to improve the signal-to-noise ratio and to establish repeatability^46^. The bias voltages reported in Figs 5 and 6 are not exactly equivalent, since the data for each were collected using different samples and measurement apparatus. When the devices were operated using a voltage source within the regions of instabilities, we observed a diffuse high-temperature channel (Fig. 6a, 0.75 V) that appeared to transform into two adjacent channels at higher voltage (Fig. 6c, 0.80 V). A further voltage increase was accompanied by extinguishing the original channel and a temperature increase in the second channel (Fig. 6f, 0.85 V). This cross-over from one channel to another is consistent with that expected from the results in Fig. 5e (a transition from the α*–*λ decomposition to the γ–λ decomposition, causing a new channel to form).

## Discussion

The experimental observations of (a) voltage-sourced electroforming of a non-volatile conducting channel in TaO_x_ memristors (Fig. 2), (b) current-density channel and electric-field fragment formation in VO_2_ (Fig. 4), and (c) current-density channel formation in NbO_2_ (Fig. 6) are all the result of instability-induced decompositions. The temperature image in Fig. 6a at 0.75 V clearly contains more noise or higher amplitude fluctuations than the other images, despite the lower applied voltage and identical measurement procedures. Since local activity can amplify thermal fluctuations (Fig. 5c), this could be a direct image of amplified local temperature variations upon the system entering a region of instability represented by region I in Fig. 5d. Thus, our proposed resolution of the debate on the cause of state transitions^12,15,16,18,19^ such as resistance switching and electronic domain decomposition is that nonlinear activated transport and instabilities provide a feedback mechanism to amplify random thermal fluctuations that trigger spontaneous symmetry-breaking and drive the system to its lowest internal energy state, which would occur even for structurally uniform and defect-free systems. Inhomogeneities (in the material composition, film thickness, etc.) may provide nucleation sites for a high current density channel. For example, the more prominent hot region observed in Fig. 6a may have nucleated at a defect. Another interesting observation is that oxides such as TaO_x_ and HfO_x_ undergo electroforming to create chemically differentiated conduction channels that support non-volatile resistance switching, but oxides such as NbO_2_ crystallize under significant Joule heating but otherwise only display reversible NDR even when voltage-driven. We attribute this difference to the activation energies for creation of oxygen defects^38,39,47^, which are relatively low in oxides such as HfO_x_ but are much larger in NbO_x_.

This model of how an electronic device can minimize its internal energy via amplified fluctuations, symmetry breaking and decomposition is completely missing from conventional numerical multi-physics simulators. The best that they can do is approximate the unstable state in a region of NDR or other electronic instability. For nanometer scale devices with increasing nonlinearity and thermal fluctuation amplitudes^4^, especially those that exhibit local activity, the results obtained by simply integrating coupled field equations can be qualitatively incorrect. Inhomogeneous and asymmetric current-density and electric-field configurations can be stabilized by space-charge regions^8^, as long as the energy required to form them is less than the energy gained through decomposition (Supplementary Note 1). Not understanding the consequences of current-density decomposition in the design of an electronic device can lead to unanticipated faults and poor reliability in an integrated circuit. However, introducing temperature fluctuations into full scale Monte Carlo or molecular-dynamics-style simulations with a uniform initial state and iterating until a lowest internal energy steady state of an electronic device has been found will likely require very long computation times, especially to generate current–voltage characteristics with high fidelity. One way to significantly cut execution times and computation costs would be to use the methods described above to map out the parameter space and use a decomposed state as the initial condition for a full physics simulation. For the interested reader, we provide an extended but preliminary analysis of the free energy of the system in Supplementary Note 2 and Supplementary Figs 16–18.

In conclusion, we performed nonlinear dynamical analysis of decompositions arising from electronic instabilities such as current-controlled NDR and Mott-transition instabilities, and showed that both current-density and electric-field decompositions are possible depending on the operating conditions and the type of instability. The decomposed states were shown to have a lower internal energy than corresponding uniform unstable steady states. As noted by Landauer^10,15^, thermodynamic minimization constraints can have a significant impact on the numerical simulations of any electronic device and are especially important for accurate modeling of nonlinear devices. We also provided experimentally obtained thermal images of such decompositions, directly revealing the formation of current-density channels and electric-field domains. We further make the connection among local activity, amplification of thermal fluctuations, and electronic instabilities, which together cause decompositions to occur.

## Methods

### Film growth and device fabrication

The TaO_x_ was deposited using ion beam induced reactive sputter deposition^38^ (using an Oxford Instruments Ionfab 300Plus machine) from a target of Ta_2_O_5_. VO_2_ was grown by high-temperature annealing of an evaporated V film in an oxygen environment^40^. NbO_2_ was grown by reactive sputter deposition^32^ from a target of NbO_2_. The compositions and film structure were studied using a variety of techniques including x-ray absorption spectroscopy (Supplementary Fig. 13), transmission electron microscopy and electron diffraction (Supplementary Fig. 15), etc. The electrodes in all cases were lithographically defined and deposited using evaporation of Pt. Detailed analyses are presented elsewhere (see the preceding references).

### Electrical measurements

Quasi-static current–voltage behavior (Fig. 2) was measured using an Agilent B1500 parameter analyzer and a Cascade probe station. The parameter analyzer was controlled through a General Purpose Interface Bus (GPIB) using software programs written in Igor. Dynamical electrical measurements (to obtain data in Fig. 6) were carried out using a custom-made circuit board capable of producing controlled and synchronized pulsed voltages that is described elsewhere^48^.

### Physical measurements

The oxygen concentration map in Fig. 2b was obtained using a scanning transmission x-ray microscope at the synchrotron at the Advanced Light Source at Lawrence Berkeley National Laboratory, beamline 11.0.2 at the oxygen K-edge. The devices for this experiment were fabricated on top of 200 nm of freely suspended silicon nitride membranes, to enable x-ray transmission in the oxygen K-edge. The details of the measurement can be found elsewhere^38,49^. The temperature maps in Fig. 4 were obtained using blackbody emission microscopy in the infrared wavelength, using InfraScope^50^, emissivity-calibrated to a temperature resolution of about 1 K and spatial resolution of about 1.5 µm. The temperature maps in Fig. 6 were obtained using thermoreflectance microscopy^46^, with a temperature resolution of about 1 K and spatial resolution of less than 0.5 µm. To enable a higher signal-to-noise ratio, we utilized a time-multiplexed technique to average the signal over synchronously repeated electrical operation^48^.

### Latent heat for the Mott transition

The latent heat for the Mott transition was accounted for by introducing a smoothed increase in ${\it{C}}_{{\mathrm{th}}}^{{\mathrm{act}}}$ over the transition temperature range, such that the area within the increase in ${\it{C}}_{{\mathrm{th}}}^{{\mathrm{act}}}$ in a plot of ${\it{C}}_{{\mathrm{th}}}^{{\mathrm{act}}}$ vs. *T* is equal to the latent heat. ${\it{C}}_{{\mathrm{th}}}^{{\mathrm{act}}}$ was calculated by using literature values for the intrinsic heat capacity and the volume of the oxide within the device. Since the rest of the device structure is unaltered during the Mott transition, we consider the thermal capacitance of only the Mott insulator within the device. The magnitude of increase in ${\it{C}}_{{\mathrm{th}}}^{{\mathrm{act}}}$ during the Mott transition depends on the width of the transition (in temperature), which in turn depends on several factors including purity of the material, crystal quality, etc. There is a wide range of transition widths and corresponding changes in heat capacities reported in the literature. The choice of the transition width does not affect our conclusions^51–53^.

For VO_2_, (in WsK^−1^)

6 $${\it{C}}_{{\mathrm{th}}}^{{\mathrm{act}}} = 1 \times 10^{ - 13} - 1250 \times 10^{ - 13}\,{\mathrm{erf}}\left( {1 + 5(340 - {\it{T}})} \right) \\ \quad + 1250 \times 10^{ - 13}\,{\mathrm{erf}}(1 + 5(340.02 - {\it{T}}))$$

For NbO_2_, (in WsK^−1^)

7 $${\it{C}}_{{\mathrm{th}}}^{{\mathrm{act}}} = 4.9 \times 10^{ - 13} - 500 \times 10^{ - 13}\,{\mathrm{erf}}\left( {1 + 5(1069.2 - {{T}})} \right) \\ \quad + 500 \times 10^{ - 13}\,{\mathrm{erf}}(1 + 5(1070.9 - {{T}}))$$

### Eliminating the possibility of ternary decomposition

Let us suppose from Fig. 5 that a total current in unstable steady-state *β* is decomposed among three stable steady-states α, γ and λ. This can be represented as

8a $${\it{x}}.{\it{j}}_{\it{\lambda }} + {\it{y}}.{\it{j}}_{\it{\gamma }} + (1 - {\it{x}} - {\it{y}}).{\it{j}}_{\it{\alpha }} = {\it{j}}_{\it{\beta }}$$

8b $${\it{x}}.({\it{j}}_{\it{\lambda }} - {\it{j}}_{\it{\alpha }}) + {\it{y}}.({\it{j}}_{\it{\gamma }} - {\it{j}}_{\it{\alpha }}) = ({\it{j}}_{\it{\beta }} - {\it{j}}_{\it{\alpha }})$$

8c $${\it{y}} = \frac{{({\it{j}}_{\it{\beta }} - {\it{j}}_{\it{\alpha }}) - {\it{x}}.({\it{j}}_{\it{\lambda }} - {\it{j}}_{\it{\alpha }})}}{{({\it{j}}_{\it{\gamma }} - {\it{j}}_{\it{\alpha }})}}$$

where *x* and *y* are the fractions of the area carrying current densities ${\it{j}}_{\it{\lambda }}$ and ${\it{j}}_{\it{\gamma }}$, respectively. The enthalpy change from the ambient for the hypothetical ternary decomposition can be calculated as

9 $${\mathrm{\Delta }}{\it{H}}_{{\mathrm{tri}}} = {\it{x}}.({\it{C}}_{{\mathrm{th}}}^{{\mathrm{act}}}.({\it{T}}_{\it{\lambda }} - {\it{T}}_{{\mathrm{amb}}}) + {\mathrm{\Delta }}{\it{H}}_{{\mathrm{Mott}}}) + {\it{y}}.{\it{C}}_{{\mathrm{th}}}^{{\mathrm{act}}}.({\it{T}}_{\it{\gamma }} - {\it{T}}_{{\mathrm{amb}}})\\ \quad + (1 - {\it{x}} - {\it{y}}).{\it{C}}_{{\mathrm{th}}}^{{\mathrm{act}}}.({\it{T}}_{\it{\alpha }} - {\it{T}}_{{\mathrm{amb}}})$$

where ${\it{T}}_{\it{\lambda }}$, ${\it{T}}_{\it{\gamma }}$ and ${\it{T}}_{\it{\alpha }}$ represent the temperatures corresponding to ${\it{j}}_{\it{\lambda }}$, ${\it{j}}_{\it{\gamma }}$ and ${\it{j}}_{\it{\alpha }}$, respectively.

Substituting Eq. (8c) in Eq. (9),

10 $$\begin{array}{l}{\mathrm{\Delta }}{\it{H}}_{{\mathrm{tri}}} = {\it{x}}.({\it{C}}_{{\mathrm{th}}}^{{\mathrm{act}}}.({\it{T}}_{\it{\lambda }} - {\it{T}}_{{\mathrm{amb}}}) + \Delta {\it{H}}_{{\mathrm{Mott}}})\\ \quad \quad \quad + \frac{{({\it{j}}_{\it{\beta }} - {\it{j}}_{\it{\alpha }}) - {\it{x}}.({\it{j}}_{\it{\lambda }} - {\it{j}}_{\it{\alpha }})}}{{({\it{j}}_{\it{\gamma }} - {\it{j}}_{\it{\alpha }})}}.{\it{C}}_{{\mathrm{th}}}^{{\mathrm{act}}}.({\it{T}}_{\it{\gamma }} - {\it{T}}_{{\mathrm{amb}}})\\ \quad \quad \quad + \left( {1 - {\it{x}} - \frac{{({\it{j}}_{\it{\beta }} - {\it{j}}_{\it{\alpha }}) - {\it{x}}.\left( {{\it{j}}_{\it{\lambda }} - {\it{j}}_{\it{\alpha }}} \right)}}{{\left( {{\it{j}}_{\it{\gamma }} - {\it{j}}_{\it{\alpha }}} \right)}}} \right).{\it{C}}_{{\mathrm{th}}}^{{\mathrm{act}}}.({{T}}_{\mathrm{\alpha }} - {\it{T}}_{{\mathrm{amb}}})\end{array}$$

which is a linear function of *x* and therefore the minimum value of ${\mathrm{\Delta }}{{H}}_{{\mathrm{tri}}}$ will appear at *x* = 0 or *x* = 1. Both cases eliminate one of the three components of the ternary decomposition, and hence the most stable configuration is a binary decomposition.

### Data availability

Additional data, if any, which support the findings of this study are available from the authors upon reasonable request.

## Electronic supplementary material

Supplementary Information
