# Supplementary material for: Separation of current density and electric field domains caused by nonlinear electronic instabilities
Source: Nat Commun. 2018 May 23;9:2030. doi: 10.1038/s41467-018-04452-w (PMC5966426; doi:10.1038/s41467-018-04452-w)
Supplement: Supplementary file 1 — Supplementary Information [file 41467_2018_4452_MOESM1_ESM.pdf]

Separation of Current Density and Electric Field Domains Caused by Nonlinear Electronic Instabilities

Suhas Kumar and R. Stanley Williams

Hewlett Packard Labs, 1501 Page Mill Rd, Palo Alto, CA 94304, USA

Supplementary Information

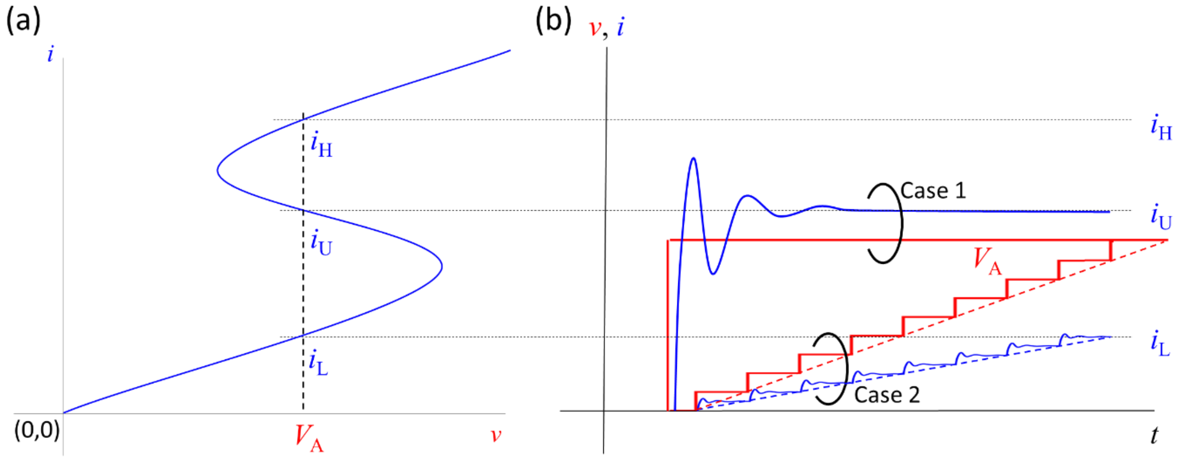

Supplementary Figure 1: Experimentally accessing the unstable part of an NDR *i*-*v* curve via transients (illustration). (a) Illustration of a current-voltage behavior with NDR. An applied voltage V_A, along with three possible current levels (i_H, i_L and i_U) are marked. (b) Case 1: V_A applied instantly in a single step (red), and the resulting current (blue). The initial transient variation in the current caused by inevitable capacitance in the system can be significant enough for the system to overshoot i_L and settle into the unstable steady state i_U. Subsequently, this unstable state will decompose into two domains characterized by current densities characteristic of the neighboring stable steady states. Case 2: V_A applied over multiple sequential steps (red), and the resulting current (blue). Since the voltage amplitude at any given step is much smaller than in Case 1, the transient current levels are significantly smaller in magnitude, and hence are not sufficient to overshoot the lowest steady state. Upon reaching V_A, the system settles into i_L. Thus, transients following a step voltage can access unstable steady states in a system, whereas a voltage sweep cannot access the unstable part of the *i*-*v* curve. The dashed red and blue lines in Case 2 represent the inferred experimental measurement during a quasi-dc voltage sweep.

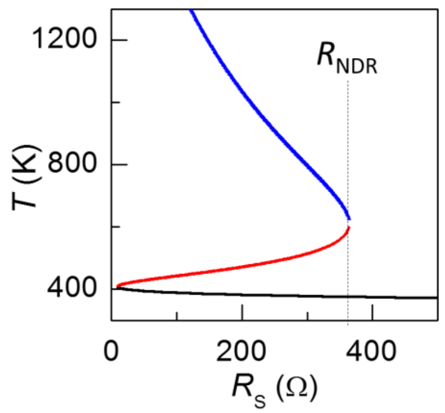

Supplementary Figure 2: Stabilization criteria for R_S. Steady-state temperatures plotted against R_S calculated using the procedure described in main manuscript Fig. 1 for the maximum V_{ext} for which NDR was observed, if any. Stable (black and blue) steady-states and unstable (red) steady-states are plotted. The minimum value of R_S required to stabilize NDR is equal to R_{NDR}. In all calculations, an internal electrode resistance of ~100 Ω was assumed.

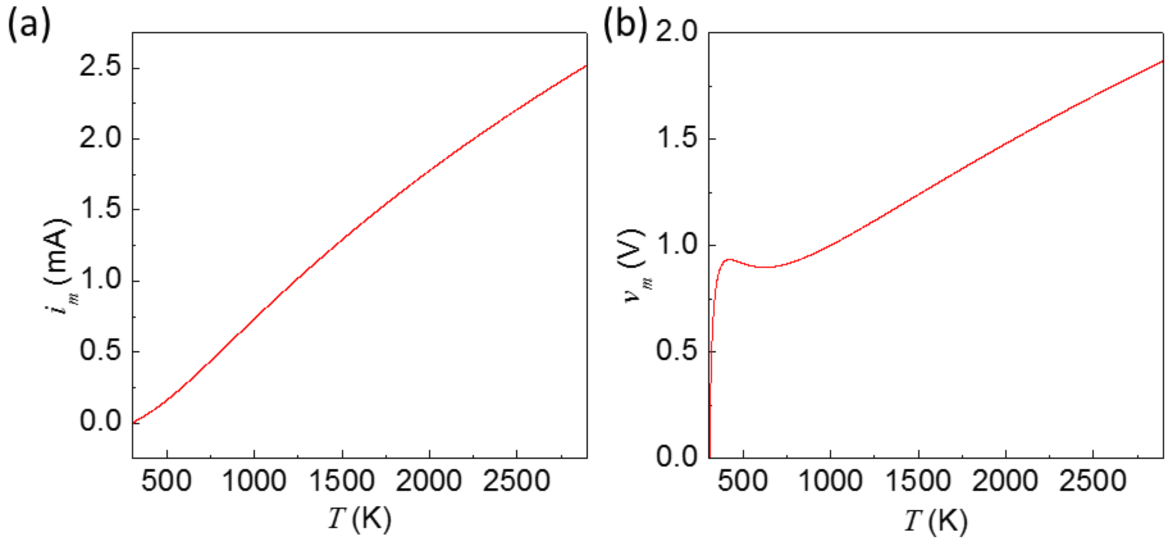

Supplementary Figure 3: Temperature-dependence of current and voltage in main manuscript Fig. 1. (a) i_m vs. T for the data corresponding in main manuscript Fig. 1. (b) v_m vs. T for the data corresponding in main manuscript Fig. 1.

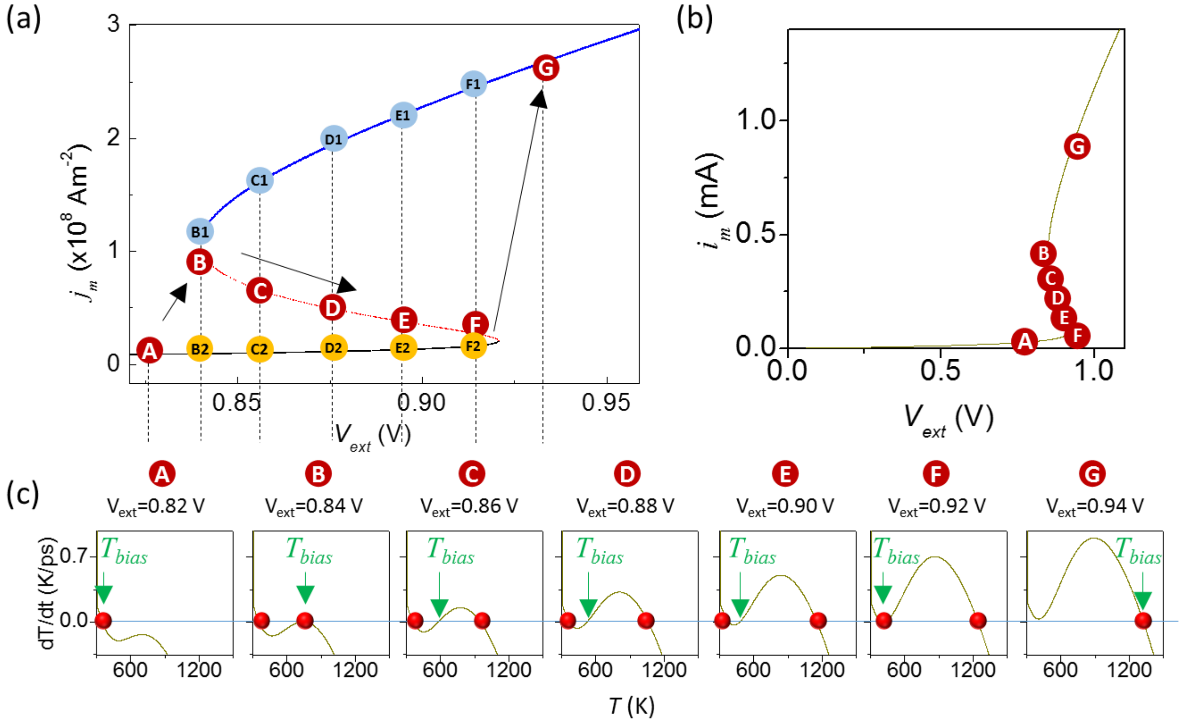

Supplementary Figure 4: Illustration of evolution of decomposition. (a) j_m vs. V_{ext} (same data as main manuscript Fig. 1f). 'A'-'G' are bias points on this curve. 'B1'-'F1' and 'B2'-'F2' are decomposed current densities corresponding to biases 'B'-'F'. Black arrows indicate the route from 'A' to 'G'. (b) Approximate positions of 'A'-'G' on the i_m vs. V_{ext} plot with R_S=0. (c) dT/dt vs. T corresponding to the different V_{ext} applied (for the biases 'A'-'G'). Red spheres indicate the stable steady-states, while the green arrow indicates the temperature bias, corresponding to the current bias.

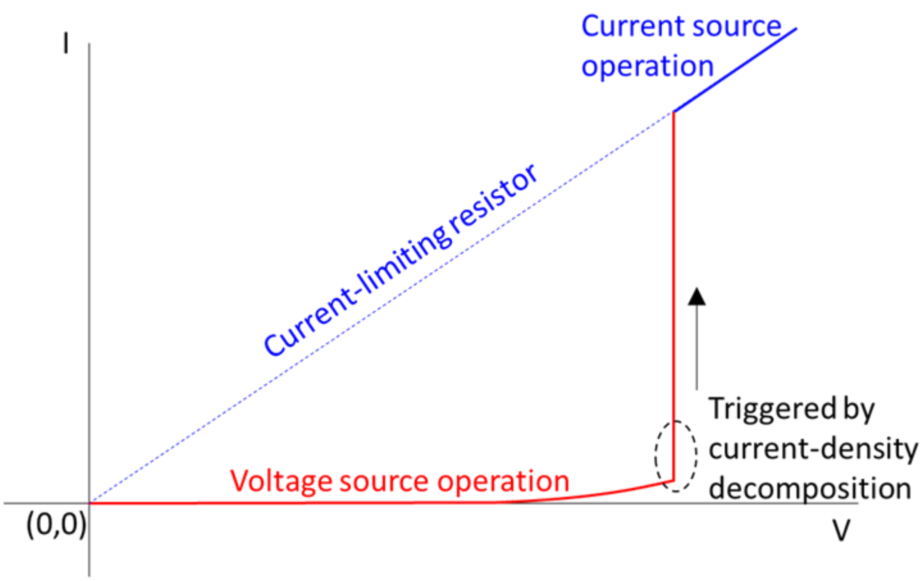

Supplementary Figure 5: Electroforming of resistive switching by current-density decomposition. Schematic current-voltage curve during the first non-volatile resistance change of a memristor driven by a voltage source. The circuit contains an additional series resistor to limit the current (represented by the dashed blue line) during the transition from the high-resistance state to a low-resistance state. In the high-resistance state (solid red curve), the additional series resistance is negligible compared to the memristor resistance, whereas in the low-resistance state (solid blue curve), the series resistance is higher than the memristor resistance. In the high-resistance state, nearly all the source voltage drops across the memristor, which is thus voltage driven. However, in the low-resistance state, since the series resistance dominates, the memristor is operated by a voltage source in series with a larger series resistor, which is essentially a current source. Thus, the electroforming operation causes a current-density decomposition, producing a high current and temperature channel that undergoes physical and chemical changes.

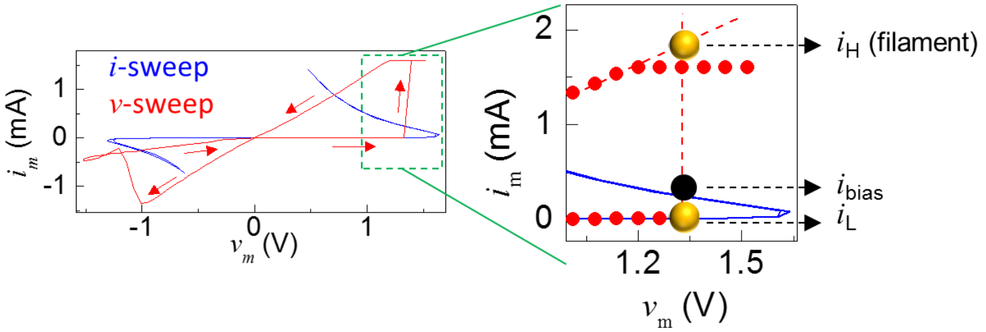

Supplementary Figure 6: Identifying the stable and unstable current levels in main manuscript Fig. 2a. (a) Reproduction of data from main manuscript Fig. 2a, along with a magnified vide of the region used to choose the three different current levels. Since the blue curve crosses the red curve at about 0.6 V, there was some difference in the device configuration between the two curves. During electroforming, due to several material and interfacial effects, there was an additional series resistance added to the device (so the new electrode resistance was higher than the virgin electrode resistance). Since our data is reported for steady state, we consider the new value of electrode resistance. This is why, to match our experimental conditions, we chose i_H as that limited by the device.

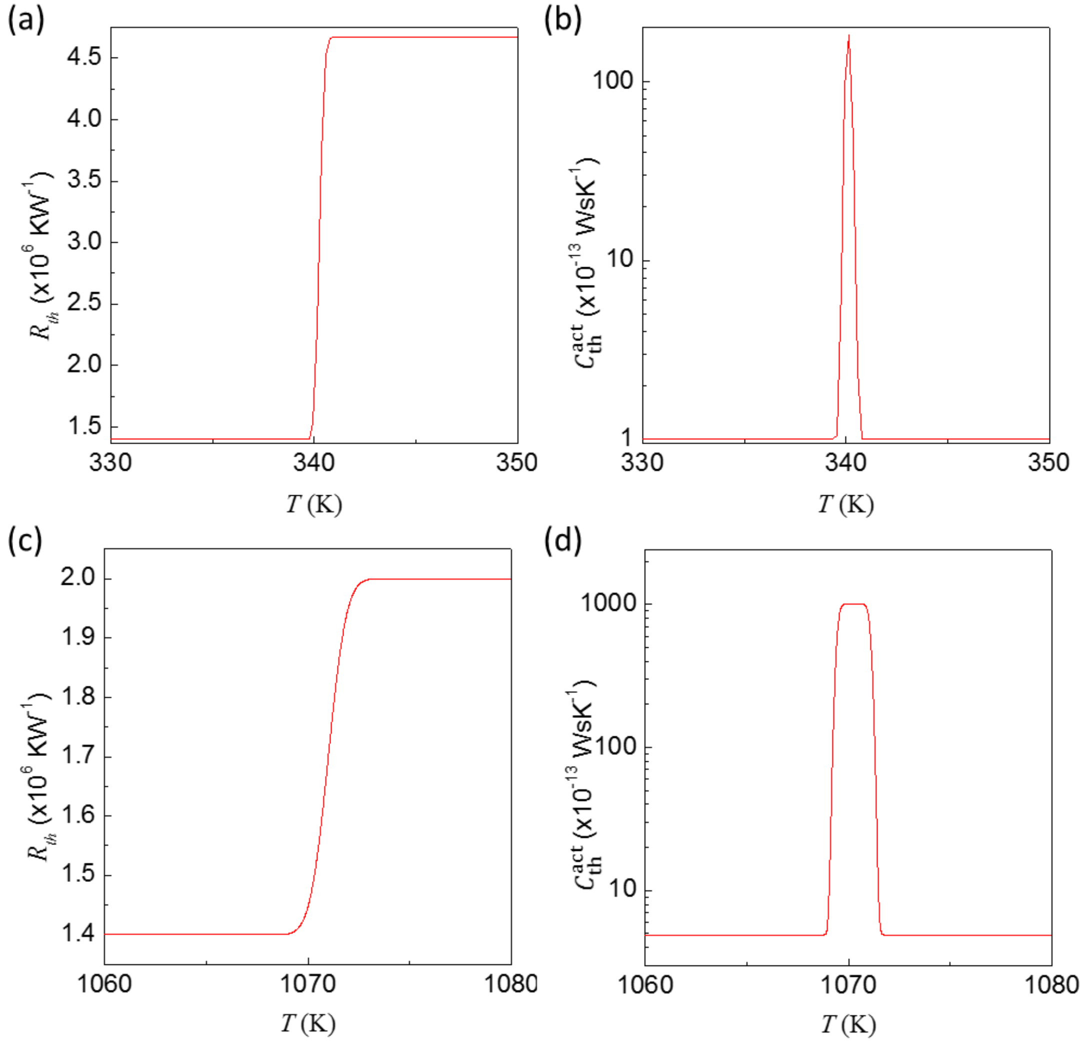

Supplementary Figure 7: Parameterizing latent heat within C_{th}^{act} and R_{th} for the Mott transition. (a) R_{th} as a function of T and (b) C_{th}^{act} as a function of T to represent VO_2, with T_{MIT} of 340 K. (c) R_{th} as a function of T and (d) C_{th}^{act} as a function of T to represent NbO_2, with T_{MIT} of 1070 K.

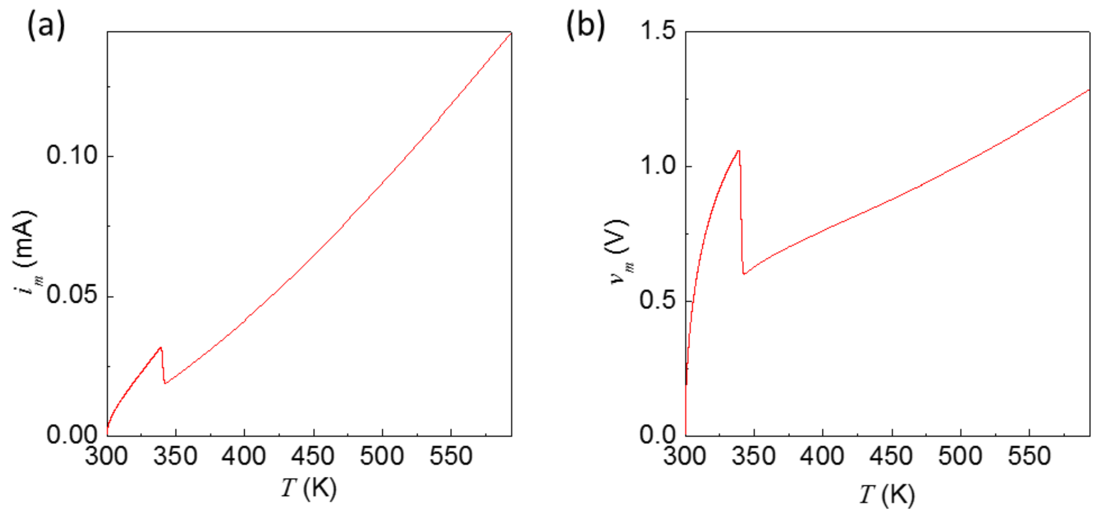

Supplementary Figure 8: Extended data of main manuscript Figure 3. (a) i_m vs. T for the data corresponding in main manuscript Fig. 3. (b) v_m vs. T for the data corresponding in main manuscript Fig. 3.

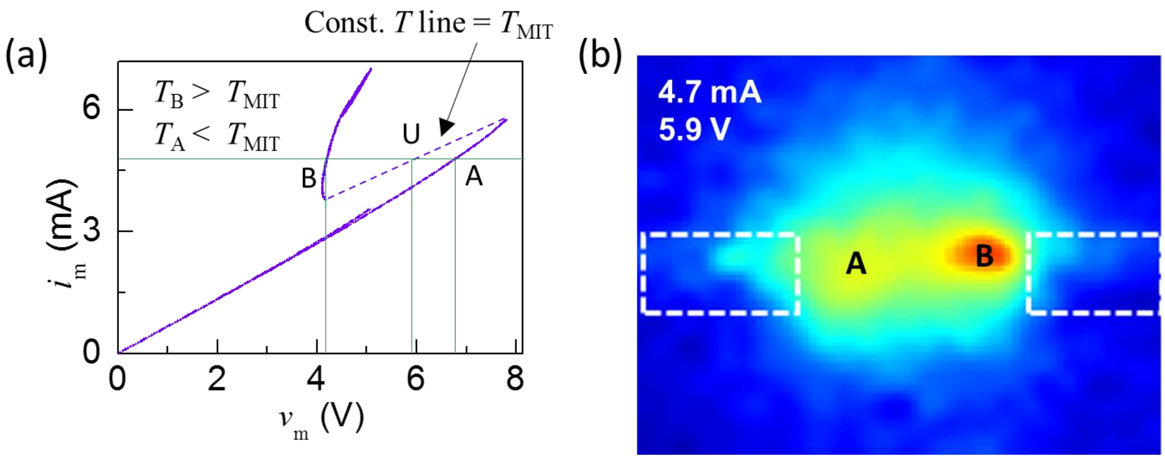

$i_m = 4.7$ mA

$v_A = 6.8$ V; $v_U = 5.9$ V; $v_B = 4.2$ V;

$T_A < T_U < T_B$

$T_U = T_{MIT}$

Supplementary Figure 9: Counter-intuitive correspondence between power dissipation and temperature. (a) Current-voltage plot displayed in main manuscript Fig. 4, with a dashed line representing the constant temperature (T_{MIT}), following the model described by main manuscript Fig. 3a. For operation using a current source of 4.7 mA, there are three steady-state voltages possible – v_A, v_U and v_B, such that $v_A > v_U > v_B$. 'A' and 'B' are stable steady-states, while 'U' is an unstable steady-state. Temperatures corresponding to these steady-states are T_A, T_U and T_B, such that $T_A < T_U < T_B$, with $T_U =$

T_{MIT}. (b) A temperature map obtained by applying a constant current from a current source (reproduced from main manuscript Fig. 4), with field domains 'A' and 'B' marked, corresponding to the stable steady-states 'A' and 'B' in (a). The temperature of a domain with a lower power dissipation ('B') being higher, while that of the domain with a higher power dissipation ('A') being lower, is a counter-intuitive observation being highlighted here.

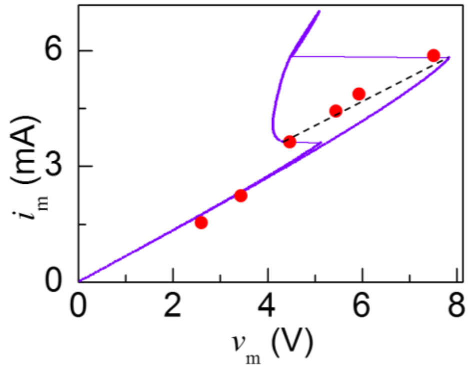

Supplementary Figure 10: Experimentally measuring the unstable part of the *i*-*v* curve. Current-voltage data obtained from the measurements shown in main manuscript Figs. 4c-4h (solid red circles) over the data shown in main manuscript Fig. 4a. The dashed black line represents the unstable part of the *i*-*v* curve inferred from modeling.

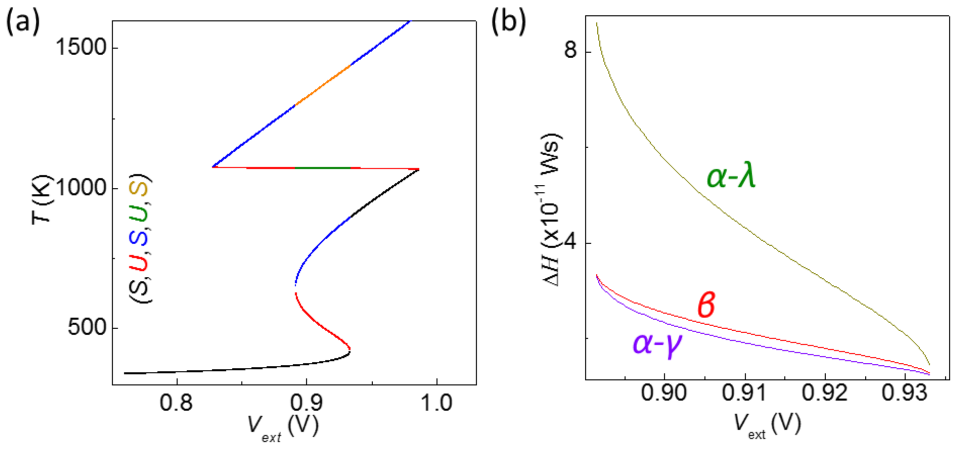

Supplementary Figure 11: Extended data of main manuscript Fig. 5. (a) T vs. V_{ext} for the data corresponding in main manuscript Fig. 5. Stable ('S') and unstable ('U') steady-states are color-coded with the legend. (b) ΔH vs. V_{ext} for a total current corresponding to β in main manuscript Fig. 5. Two possible decomposed configurations (α-λ) and (α-γ) along with the unstable state (β) are displayed.

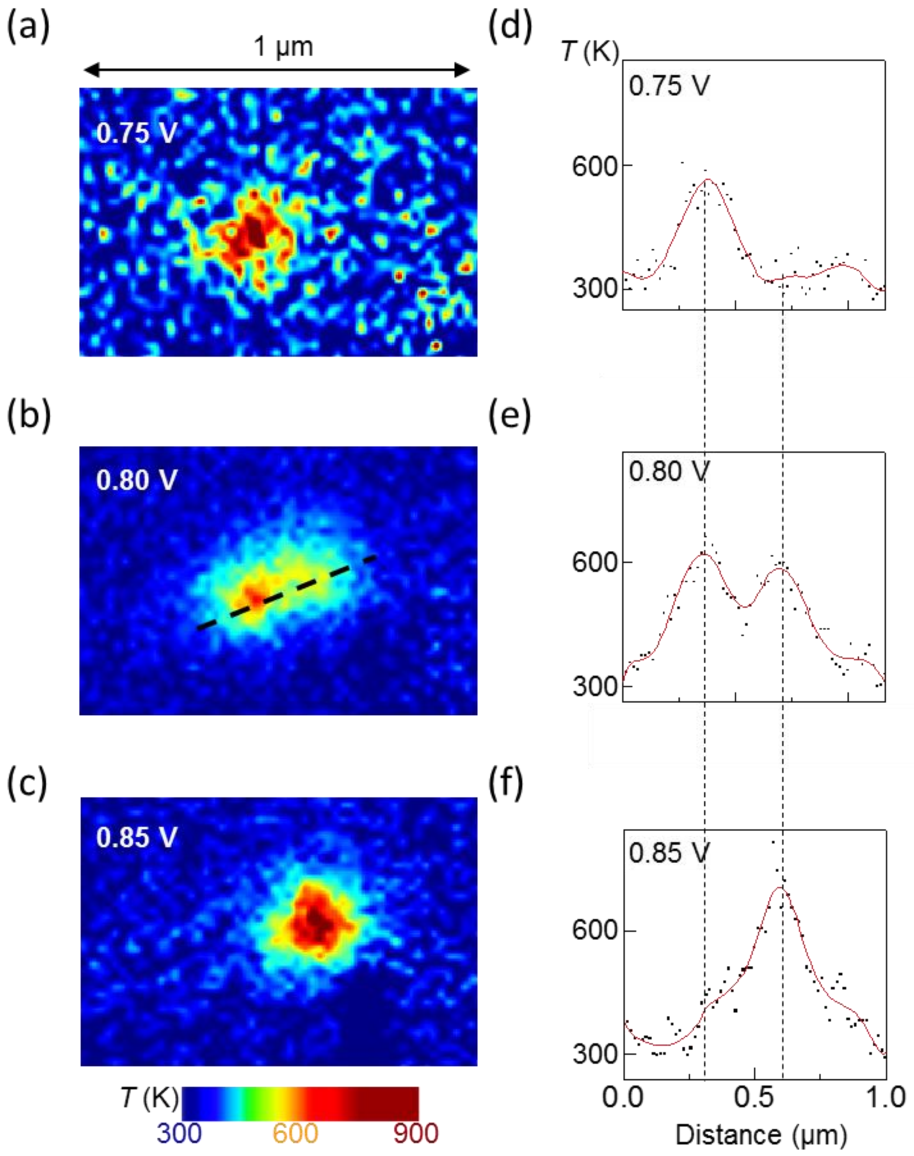

Supplementary Figure 12: Extended data of main manuscript Fig. 6. (a)-(c) Magnified temperature maps of main manuscript Figs. 6a-6c. Black dashed line in the map corresponding to '0.80 V' represents the region of 1-dimensional cross section displayed in (d)-(f) (same as main manuscript Figs. 6d-6f).

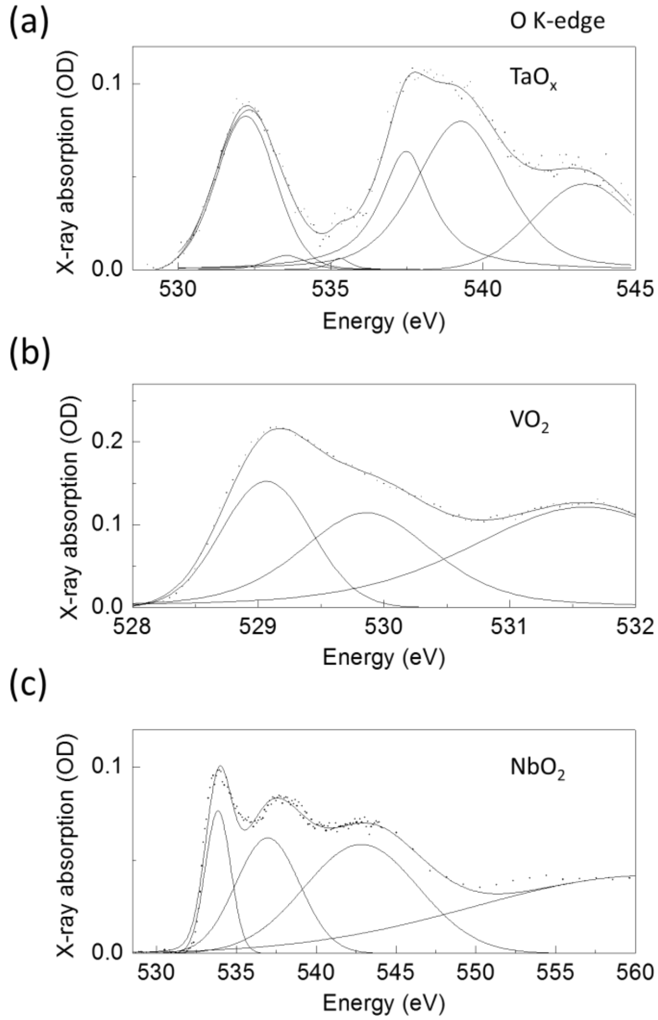

Supplementary Figure 13: As-grown TaO_x, VO_2 and NbO_2. O K-edge absorption spectra of as-grown (a) TaO_x, (b) VO_2 and (c) NbO_2 used in this study. The prominent bands of each of the oxides are clearly visible and identifiable.[1-3]

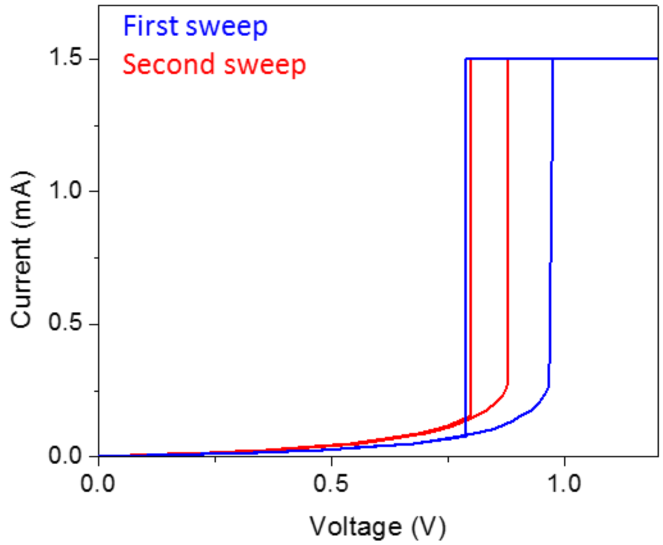

Supplementary Figure 14: Current-voltage behavior of NbO_2 measured using a voltage source. A current limit of 1.5 mA was required while collecting this data to prevent the device from being destroyed by Joule heating. The resulting volatile threshold switching is shown. The first sweep required a slightly higher voltage compared to the subsequent repeatable sweeps and likely produced irreversible material changes. This data was obtained on a micrometer-sized crosspoint device. By comparing this result to the data obtained using a current source in main manuscript Fig. 5a, we can see that both the current-controlled NDR and the Mott-transition-driven instability are contained within the voltage-sweep pinched hysteresis loop, which is a fingerprint of a memristor.

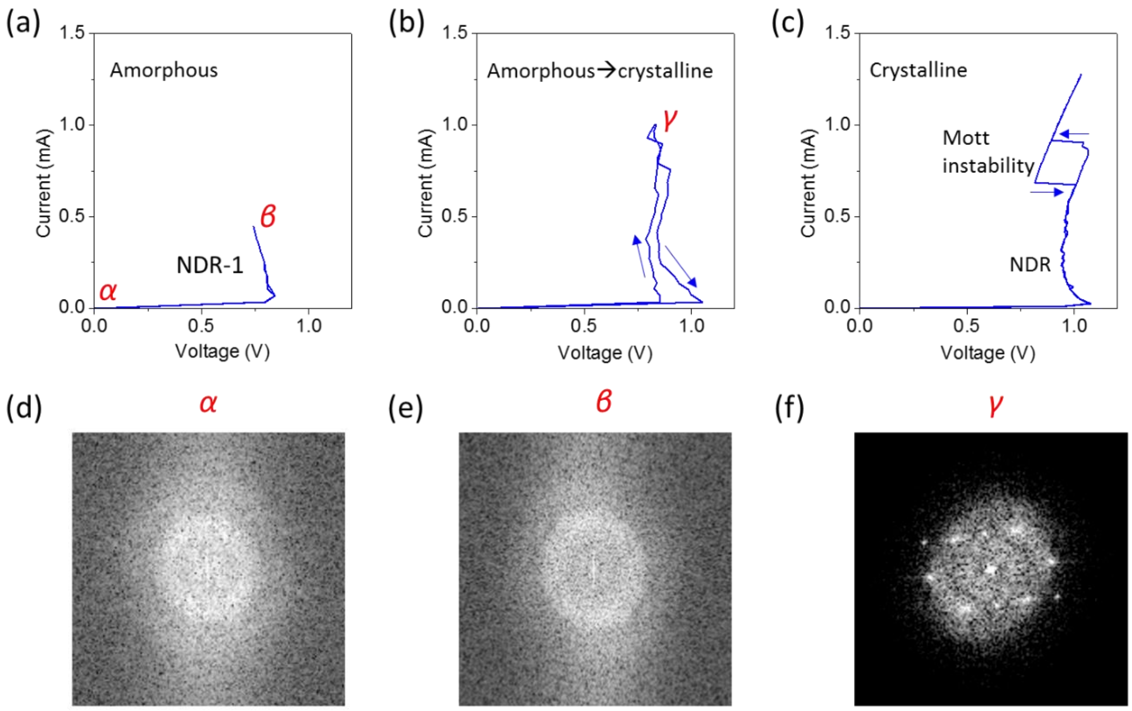

Supplementary Figure 15: NDR observed in amorphous and crystalline NbO_2. (a) Repeatable current-voltage curve of a device that has not been subjected to current above those required to cause NDR-2. These current levels also did not cause irreversible crystallization of the NbO_2 layer. Thus, the NDR-1 (current-controlled NDR) observed here represents the behavior of the amorphous NbO_2. (b) Current-voltage curve of the same device in (a) upon the first instance of subjecting it to current levels above those required for NDR-2. The sharp changes (that were irreproducible and irreversible) are most likely associated with the irreversible crystallization that was confirmed by electron microscopy. (c) Repeatable current-voltage plot of the same device after the behavior observed in (b). The NDR-1 and NDR-2 observed here represent the behavior of the crystalline NbO_2. (d)-(f) Electron diffraction patterns obtained from a transmission electron microscope on three different devices that were subject to different current levels, using a current-source – α: no applied current, β: an applied current sufficient to cause a current-controlled NDR, but insufficient to cause a Mott-transition-driven instability, and γ: an applied current that was sufficient to cause a Mott-transition-driven instability. The cases for α and β display no clear diffraction spots, indicating an amorphous structure, whereas the case of γ displays clear diffraction spots corresponding to a tetragonal [001] crystal projection of NbO_2.

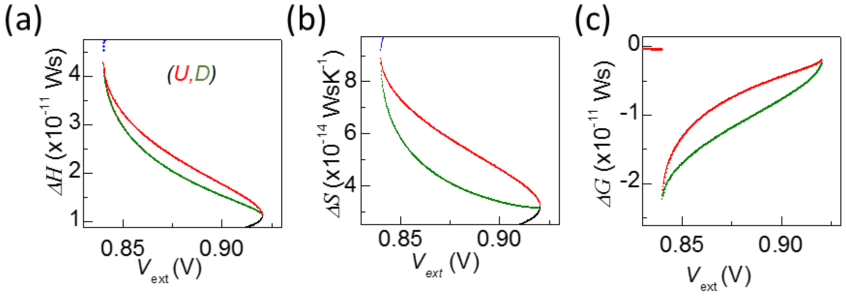

Supplementary Figure 16: Free energy of decomposition during current-controlled NDR. (a) ΔH vs. V_{ext} for the unstable ('U') and decomposed ('D') configurations during current-controlled NDR (reproduced from main manuscript Fig. 1i). (b) Corresponding ΔS, and (c) ΔG.

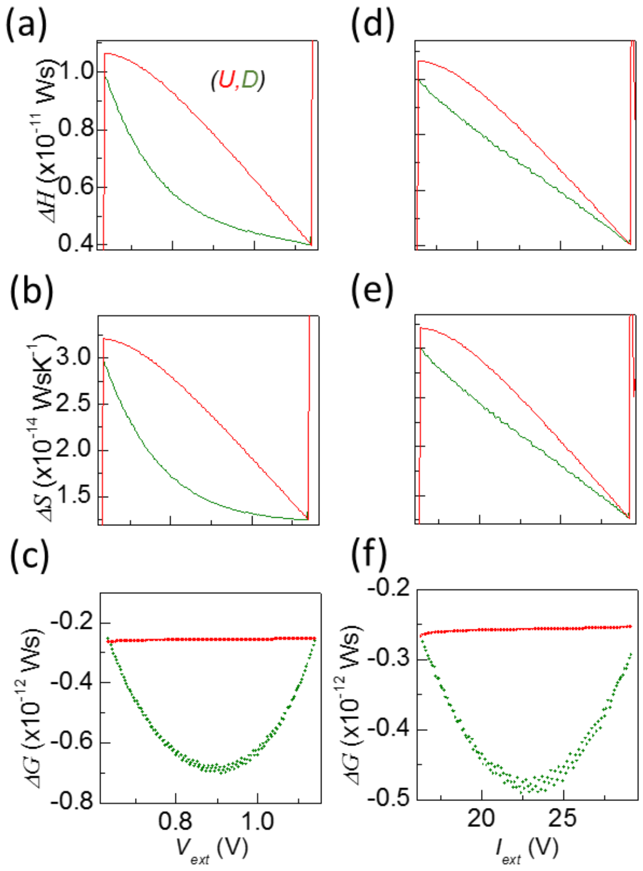

Supplementary Figure 17: Free energy of decomposition during *T*-controlled instability. (a) *ΔH* vs. V_{ext} for the unstable ('U') and decomposed ('D') configurations during current-controlled NDR (reproduced from main manuscript Fig. 1i). (b) Corresponding *ΔS*, and (c) *ΔG*. (d)-(f) Plots of *ΔH, ΔS* and *ΔG* obtained for a current source (I_{ext}) operation. (a) and (d) are reproduced from main manuscript Figs. 3h and 3i.

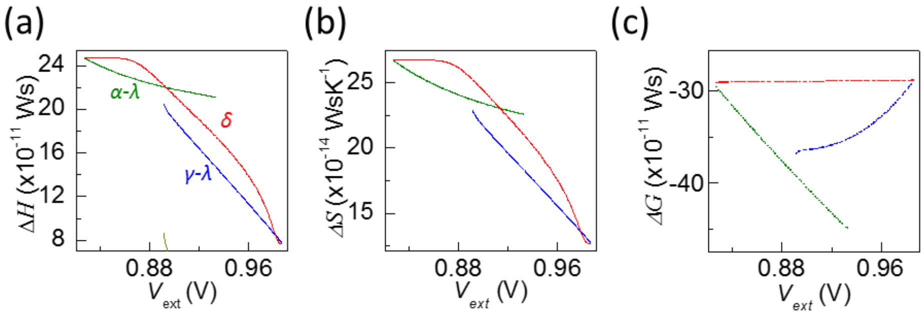

Supplementary Figure 18: Free energy of decomposition during dual instabilities. (a) ΔH vs. V_{ext} for the unstable ('U') and decomposed ('D') configurations during dual instabilities (reproduced from main manuscript Fig. 5e). (b) Corresponding ΔS, and (c) ΔG.

Supplementary Note 1: Relative to ΔH, capacitive energy change from possible charge separations contributes negligibly to internal energy

A possible significant contribution to the internal energy, apart from the enthalpy increase from the ambient conditions, could come from charge separations or formation of space charges, leading to a change in the capacitive energy stored between the electrode plates contacting the nonlinear material, such as a metal oxide. In order to make a comparison of the magnitude of this contribution to the change in internal energy, consider the following estimate for ΔH for the simple case of a temperature independent $C_{\mathrm{th}}^{\mathrm{act}}$.

$$\Delta H = C_{\mathrm{th}}^{\mathrm{act}}\Delta T = 55\ \mathrm{JK^{-1}mol^{-1}} \div 125\ \mathrm{g\ mol^{-1}} \times 6\ \mathrm{g\ cm^{-3}} \div 15\ \mathrm{nm} \times \Delta T$$

$$= 0.0396\ \mathrm{Jm^{-2}K^{-1}} \times \Delta T$$

Supplementary Equation (1)

For the capacitive energy change, let us assume the extreme possibility that the potential difference caused by the charge separations is equal to the entire voltage drop across NDR of about 1V. The resulting capacitive energy change is

$$\Delta E_{Cap} = \frac{1}{2}\frac{\epsilon_0\epsilon_r A}{d}(V_1^2 - V_2^2) = \frac{\frac{1}{2}(8.8\times10^{-12}\mathrm{Fm^{-1}}\times 20\times A)}{15\ \mathrm{nm}}((1\ V)^2 - 0) = 0.006\ \mathrm{Jm^{-2}}$$

Supplementary Equation (2)

For even a moderate temperature increase of about 70 K to cause NDR, ΔH is at least two orders of magnitude greater than ΔE_{Cap} and hence ΔH very closely represents the internal energy of the system.

Supplementary Note 2: Free energy during decomposition

While the internal energy represented by ΔH indicates an important thermodynamic difference between a system's unstable and decomposed configurations, the free energy of the system, which accounts for both internal energy and entropy, could provide additional insights. Such insights may include a measure of disorder in the different configurations, spontaneity (or the lack of) of the process of electronic

decomposition under given conditions, and the reversibility of the process. It must also be appreciated that precise estimations of several such thermodynamic quantities during decompositions involve non-trivial situations including non-equilibrium conditions, nonlinear dynamics, temporally changing physical parameters, and the effect of amplified ambient fluctuations, among several others. In this supplementary note, we provide highly simplified calculations of the free energy of the system, which are meant to identify another thermodynamic quantity that distinguishes an electronic decomposition. An in-depth examination of the thermodynamics and the nature of electronic decompositions remains a topic of interest for a dedicated study.

We calculate the free energy of the decomposed configuration (ΔG_{Dec}) of the system using Supplementary Equations 3a-3c:

$$\Delta G_{Dec} = \Delta H_{\mathrm{Dec}} - T\Delta S_{\mathrm{Dec}}$$ Supplementary Equation (3a)

$$\Delta G_{Dec} = (1-x)\Delta H_{\mathrm{L}} + x\,\Delta H_{\mathrm{H}} - T_{\mathrm{L}}\,(1-x)\Delta S_{\mathrm{L}} - T_{\mathrm{H}}\,x\,\Delta S_{\mathrm{H}}$$ Supplementary Equation (3b)

$$\Delta G_{\mathrm{Dec}} = (1-x)\left[\int_{T_{\mathrm{amb}}}^{T_{\mathrm{L}}} C_{\mathrm{th}}^{\mathrm{act}}(T)\mathrm{d}T\right] + x\left[\int_{T_{\mathrm{amb}}}^{T_{\mathrm{H}}} C_{\mathrm{th}}^{\mathrm{act}}(T)\mathrm{d}T\right] - T_{\mathrm{L}}\,(1-x)\left[\int_{T_{\mathrm{amb}}}^{T_{\mathrm{L}}} \frac{C_{\mathrm{th}}^{\mathrm{act}}(T)}{T}\mathrm{d}T\right] - T_{\mathrm{H}}\,x\left[\int_{T_{\mathrm{amb}}}^{T_{\mathrm{H}}} C_{\mathrm{th}}^{\mathrm{act}}(T)\mathrm{d}T\right]$$

Supplementary Equation (3c)

And we calculate the free energy of the unstable (ΔG_{U}) steady states using Supplementary Equations 4a-4b as:

$$\Delta G_{U} = \Delta H_{\mathrm{U}} - T\Delta S_{\mathrm{U}}$$ Supplementary Equation (4a)

$$\Delta G_{\mathrm{U}} = \left[\int_{T_{\mathrm{amb}}}^{T_{\mathrm{U}}} C_{\mathrm{th}}^{\mathrm{act}}(T)\mathrm{d}T\right] - T_{\mathrm{U}}\left[\int_{T_{\mathrm{amb}}}^{T_{\mathrm{U}}} \frac{C_{\mathrm{th}}^{\mathrm{act}}(T)}{T}\mathrm{d}T\right]$$

Supplementary Equation (4b)

Here ΔH and ΔS are changes in the internal enthalpy and entropy from the ambient to the decomposed (subscript Dec) or the unstable (subscript U) configurations, respectively; C_{th}^{act} is the thermal capacitance of only the active NbO_2 layer within the device structure; subscripts L, H and U correspond to regions with temperatures T_L, T_H and T_U, respectively, and T_{amb} is the ambient temperature (300 K).

Supplementary Figs. 16-18 display plots of ΔH, ΔS and ΔG corresponding to the three cases studied in the manuscript (current-controlled NDR, *T*-controlled instability, and dual instabilities). In all the cases, as intuition would predict, the decomposed configuration has a lower entropy relative to the homogeneous unstable configuration. Despite this, the free energy is always lower in the decomposed configuration, suggesting that a spontaneous electronic decomposition is favored, which was inferred from ΔH calculations, as discussed in the manuscript.

Supplementary References

1 Kumar, S., Pickett, M. D., Strachan, J. P., Gibson, G., Nishi, Y. & Williams, R. S. Local Temperature Redistribution and Structural Transition During Joule-Heating-Driven Conductance Switching in VO2. *Advanced Materials* **25**, 6128-6132, (2013).

2 Kumar, S., Graves, C. E., Strachan, J. P., Grafals, E. M., Kilcoyne, A. L. D., Tyliszczak, T., Weker, J. N., Nishi, Y. & Williams, R. S. Direct Observation of Localized Radial Oxygen Migration in Functioning Tantalum Oxide Memristors. *Advanced Materials* **28**, 2772-2776, (2016).

3 Soriano, L., Abbate, M., Fuggle, J. C., Jimenez, M. A., Sanz, J. M., Mythen, C. & Padmore, H. A. The O 1s x-ray absorption spectra of transition-metal oxides: The TiO2-ZrO2-HfO2 and V2O5-Nb2O5-Ta2O5 series. *Solid State Communications* **87**, 699-703, (1993).
